# Clinical benefits of intraoperative radiotherapy for the recurrence of centrally located hepatocellular carcinoma with microvascular invasion

**DOI:** 10.1002/cnr2.1928

**Published:** 2023-10-31

**Authors:** Changcheng Tao, Kai Zhang, Zonggui Tao, Yue Liu, Anke Wu, Liming Wang, Qinfu Feng, Fan Wu, Weiqi Rong, Jianxiong Wu

**Affiliations:** ^1^ Department of Hepatobiliary Surgery National Cancer Center/National Clinical Research Center for Cancer/Cancer Hospital Chinese Academy of Medical Sciences and Peking Union Medical College Beijing China; ^2^ Department of Interventional Therapy Tianjin Medical University Cancer Institute and Hospital, National Clinical Research Center for Cancer, Key Laboratory of Cancer Prevention and Therapy, Tianjin's Clinical Research Center for Cancer Tianjin China; ^3^ Department of Imaging Jinan City People's Hospital, Shandong First Medical University Jinan China; ^4^ Department of Radiation Oncology National Cancer Center/National Clinical Research Center for Cancer/Cancer Hospital, Chinese Academy of Medical Sciences and Peking Union Medical College Beijing China

**Keywords:** hepatocellular carcinoma, intraoperative radiation, microvascular invasion

## Abstract

**Background:**

Although the efficacy and safety of intraoperative radiotherapy (IORT) in the treatment of malignant tumours, such as breast cancer, have been documented, it remains unclear whether this treatment is effective for centrally located hepatocellular carcinoma (HCC) with microvascular invasion (MVI).

**Aims:**

This study aimed to explore the efficacy and safety of IORT in the treatment of centrally located HCC with MVI.

**Methods and results:**

Patients with centrally located HCC, who underwent surgery between January 2016 and January 2020, were enrolled. The patient cohort was then allocated to two groups: those who underwent IORT combined with liver resection (IORT+LR); or LR alone (LR). Propensity score matching and Cox proportional hazards regression analyses were performed. The Kaplan–Meier method was used to estimate recurrence‐free survival (RFS), and the log‐rank test was used to determine whether RFS differed between the groups. Subgroup analysis was performed to evaluate differences in RFS and early recurrence rates in patients with different MVI grades. E‐values were generated to measure the sensitivity to unmeasured confounding factors. In total, 97 patients were enrolled, 27 of whom underwent IORT+LR and 70 underwent LR alone. The 1‐, 3‐, and 5‐year RFS rates in the IORT+LR group were 66%, 50%, and 32%, respectively, whereas those in the LR group were 54%, 37%, and 26%, respectively. After matching analysis, 23 patients were successfully matched, and RFS was found to be significantly different between the two groups (*p* = .04). IORT was an independent prognostic factor for RFS (hazard ratio 0.46 [95% confidence interval 0.21–0.99]). In subgroup analysis, RFS between the IORT+LR and LR groups was significantly different in patients with MVI (M1 grade) (*p* = .0067). The postoperative early recurrence rate was significantly reduced with IORT (*p* < .05). No serious complications were reported in either group following surgery. Based on E‐values, the results appeared to be robust against unmeasured confounding factors.

**Conclusion:**

IORT+LR provided safe, feasible treatment for patients with centrally located HCC with MVI, along with an improvement in prognosis and lower early recurrence rates.

## 1 INTRODUCTION

Primary liver cancer is a prevalent form of malignant neoplasm in China, ranking third in incidence after lung and gastric cancers.^1^ Liver cancer is the second most common cause of death globally,^2^ and its morbidity rate continues to increase.^3^ Therapeutic interventions available for early stage liver cancer include surgical resection, liver transplantation, radiofrequency ablation, and transcatheter arterial chemoembolisation (TACE), with surgical resection considered the primary treatment modality.^4^ However, 70% of patients experience tumour recurrence(s).^5^

In the past, centrally located hepatocellular carcinoma (HCC) was often considered to be a contraindication to surgery due to its proximity to vital blood vessels or biliary tracts, contributing to great difficulty in surgical treatment, and high postoperative recurrence rate. Based on the conventional definition, centrally located HCC pertains to a neoplasm specifically found within Couinaud segments IV, V, and VIII of the liver.^6^ This definition does not emphasise the relationship between the tumour and its surrounding structures such as large bile ducts and blood vessels; therefore, it provides weak guidance for surgery. We present a modified interpretation in which centrally located HCC refers to liver tumour adhesion to or at a distance of <1 cm from the hepatic vein, portal vein, primary hepatic branch of the biliary system, or retrohepatic inferior vena cava, as confirmed by preoperative imaging, intraoperative macroscopic examination, and postoperative pathological examination. Furthermore, these tumours are typically found in Couinaud segments I, IV, V, and VIII or at the junction of the central segments.^7^

Owing to improvements in surgical techniques in recent years, dissemination of the concept of precise liver resection (LR), and refinements in perioperative management, surgery has become a more effective treatment for centrally located HCC. However, surgery results in a high postoperative recurrence rate if a narrow incision margin remains. Hence, it is imperative to identify an efficient and all‐encompassing treatment approach that can enhance the perioperative safety and prognosis of patients diagnosed with centrally located HCC.

Radiotherapy is a crucial therapeutic modality that has demonstrated both safety and efficacy in the management of hepatic malignancies, as substantiated by numerous previous investigations.^8^, ^9^, ^10^ The emergence of advanced radiotherapy techniques, such as three‐dimensional conformal radiotherapy, intensity‐modulated radiation therapy, and stereotactic body radiation therapy, has significantly enhanced the precision and efficacy of contemporary radiotherapy. Radiotherapy has emerged as a highly promising therapeutic modality for patients with liver cancer.^11^ Intraoperative radiotherapy (IORT) has several advantages over pre‐ and postoperative radiotherapy, such as more precise targeting, more timely treatment, shorter treatment course, and lower treatment costs. The most significant advantage of IORT is that it imparts a lower radiation hazard to other organs, such as the bowel. Recent studies have demonstrated that IORT is effective in treating breast cancer,^12^ head and neck cancer^13^ and thymoma.^14^ However, IORT requires further exploration in liver cancer.

Microvascular invasion (MVI), alternatively referred to as endovascular cancer embolus, is characterised by the presence of a tumour cell mass within the portal vein, large blood vessels, or a vascular lumen that adheres to endothelial cells.^15^ MVI is a histological feature commonly observed in HCC and is closely associated with the aggressive nature of the tumour.^16^ The presence of MVI is a significant risk factor for both overall survival (OS) and the likelihood of early relapse following surgical intervention.^17^ Previous studies have confirmed MVI using postoperative pathological examination in 11%–60% of patients with HCC who underwent surgical resection,^18^ which is consistent with our previous study, in which we reported a rate of 39%.^19^

## 2 METHODS

### 2.1 Patient selection

Data were collected from patients who underwent hepatectomies between January 2016 and January 2020. Patients were enrolled based on pre‐defined inclusion and exclusion criteria. Inclusion criteria were as follows: age ≥18 years; diagnosis of HCC and MVI confirmed through postoperative pathological examination; perform IORT; centrally located HCC adhesion to or at a distance of <1 cm from the hepatic vein, portal vein, main hepatic branch of the biliary system or retrohepatic inferior vena cava, as confirmed by preoperative imaging, intraoperative macroscopic examination and postoperative pathological examination; R0 resection; Child–Pugh class A; Eastern Cooperative Oncology Group performance status 0 or 1; and complete clinical and pathological data. Individuals with multiple liver tumours, patients who underwent radiotherapy before or after surgical resection, and those with unknown MVI classification were excluded.

The enrolment of all participants, including those who were included or excluded, was assessed by a multidisciplinary team (MDT) comprising surgeons, physicians, radiologists, pathologists, and other professionals involved in the treatment decision‐making process.

### 2.2 Treatment

#### 2.2.1 Surgical treatment

Before surgery, all patients were required undergo a liver reserve function test (indocyanine green test). Only when normal test results were obtained could patients proceed with the surgical procedure. To initiate the surgical procedure, an exploratory laparotomy was performed on the abdomen and pelvis to eliminate the possibility of distant metastases. In cases deemed necessary, intraoperative ultrasound was used to assess liver tumours. Determination of the surgical resection range considered both the entirety of the tumour and the presence of liver cirrhosis. The surgical procedure entails the application of a selective and dynamic region‐specific vascular occlusion (SDRVO) technique for personalised and accurate LR.^20^ The surgical options for treating HCC included anatomical and non‐anatomical hepatectomy.

#### 2.2.2 Intraoperative radiotherapy

In patients who intend to undergo IORT, it is imperative to validate HCC through a rapid intraoperative pathological examination. Radiation oncologists and surgeons collaborated to design the treatment strategy, considering tumour location and size, proximity to vital vessels, extent of surgical resection required, potential presence of minimal residual disease (MRD), and state of the surrounding perihepatic tissues. Factors involved in the treatment plan were also determined, including the target area, radiation dose, and electron beam applicator system used for IORT (diameter, 3.0–9.0 cm; angle, 0°, 15° or 30°). The region encompassing 1.0 cm around the tumour and a depth that could be radiated by a 90% radiation dose (range 0.5–1.5 cm, median 1.0 cm) was identified as the target area. The radiation depth was adjusted using a Bolus sheet with a thickness of 0.5 cm or 1.0 cm. Two lead plates were used to protect the perihepatic normal tissues and organs from radiation. The administration of IORT was facilitated by a mobile electron beam accelerator (Mobetron, IntraOp Medical Corporation, Sunnyvale, CA, USA) that administered a median dose of 15 Gy (range 15–17 Gy) over 3 min.

### 2.3 Follow‐up

Recurrence was defined as imaging or pathological findings suggestive of HCC. The following diagnostic procedures were routinely performed: measurement of serum α‐fetoprotein (AFP) level, liver function tests, kidney function tests, routine blood tests, and abdominal imaging via enhanced magnetic resonance imaging (MRI) or enhanced computed tomography (CT), as well as chest X‐ray. Enhanced chest CT, whole‐body bone scans, and enhanced brain MRI were performed in patients with suspected lung lesions, bone metastases, and brain lesions, respectively. The patients underwent periodic evaluations at intervals of three months during the initial two‐year period after surgical resection, followed by intervals of four to six months for the subsequent three years, and intervals of six to 12 months thereafter. Physical discomfort was assessed if the patients experienced discomfort during follow‐up. The present study was conducted up to April 2022.

This retrospective non‐interventional study, which did not hinder diagnostic or therapeutic procedures, was approved by the Ethics Committee. The findings of this research will be disseminated in the form of statistically analysed data, ensuring the exclusion of any patient‐identifying information. All relevant patient data were kept confidential, in accordance with the Declaration of Helsinki. Consent was obtained from all participants.

### 2.4 Definition and analysis

Microscopically, MVI can be classified into the following grades: M1 (low risk), ≤5 areas of MVI, all of which are ≤1 cm away from the capsule of the tumour; and M2 (high risk), >5 areas of MVI or one of which were >1 cm away from the capsule of the tumour.^21^ In this study, recurrence‐free survival (RFS) was defined as the interval between the surgical procedure and occurrence of HCC relapse.

In propensity score analysis, a certain deviation may exist between groups in a retrospective study. Matching analysis was performed to minimise selection bias between the two groups, including sex, age, AFP, MVI, HBV‐Ag, and tumour size. Propensity scores were generated by selecting variables that may influence survival.^22^ Subsequently, the two groups were matched at a ratio of 1:1, with a difference in the range of the propensity score <0.02, thus minimising selection bias between the two groups.

The patient characteristics used in the creation and dissemination of the propensity scores were evaluated by applying the standardised mean difference (SMD) both before and after the propensity score matching process.^23^ A threshold of <0.2 was considered to be acceptable. During hospitalisation, the Clavien‐Dindo grading system was used to evaluate complications. RFS in both groups was analysed before and after the matching analysis. Subgroup analysis was performed using matching analysis; more specifically, the significance of the difference in RFS between the two surgery groups was analysed in patients in the M1 and M2 subgroups separately. Recurrence time >18 months was determined to be optimal for distinguishing between early and late recurrences.^24^, ^25^ If the RFS was ≤18 months, early recurrence was considered; otherwise, late recurrence was considered.

### 2.5 Statistical methods

The statistical software packages used for all the analyses were R (http://www.R-project.org; R Foundation for Statistical Computing, Vienna, Austria) and SPSS version 23 (IBM Corporation, Armonk, NY, USA). The Kaplan–Meier method was used to estimate RFS, and the log‐rank test was used to evaluate disparity between the two groups. Prognostic factors associated with RFS were identified using univariate and multivariate Cox regression models. In the univariate analysis, multivariate analysis was conducted with variables with *p* < .1 in the univariate analysis. Variables with *p* < .05 in the multivariate analysis were deemed to be independent prognostic factors. The potential for unmeasured confounding between the two groups was investigated by computing E‐values.^26^ The E‐value serves as a metric for the magnitude of an unmeasured confounding variable that must be present to nullify the observed association between IORT and RFS. Differences with a two‐sided *p* < .05 were considered to be statistically significant.

## 3 RESULTS

**FIGURE 1:**
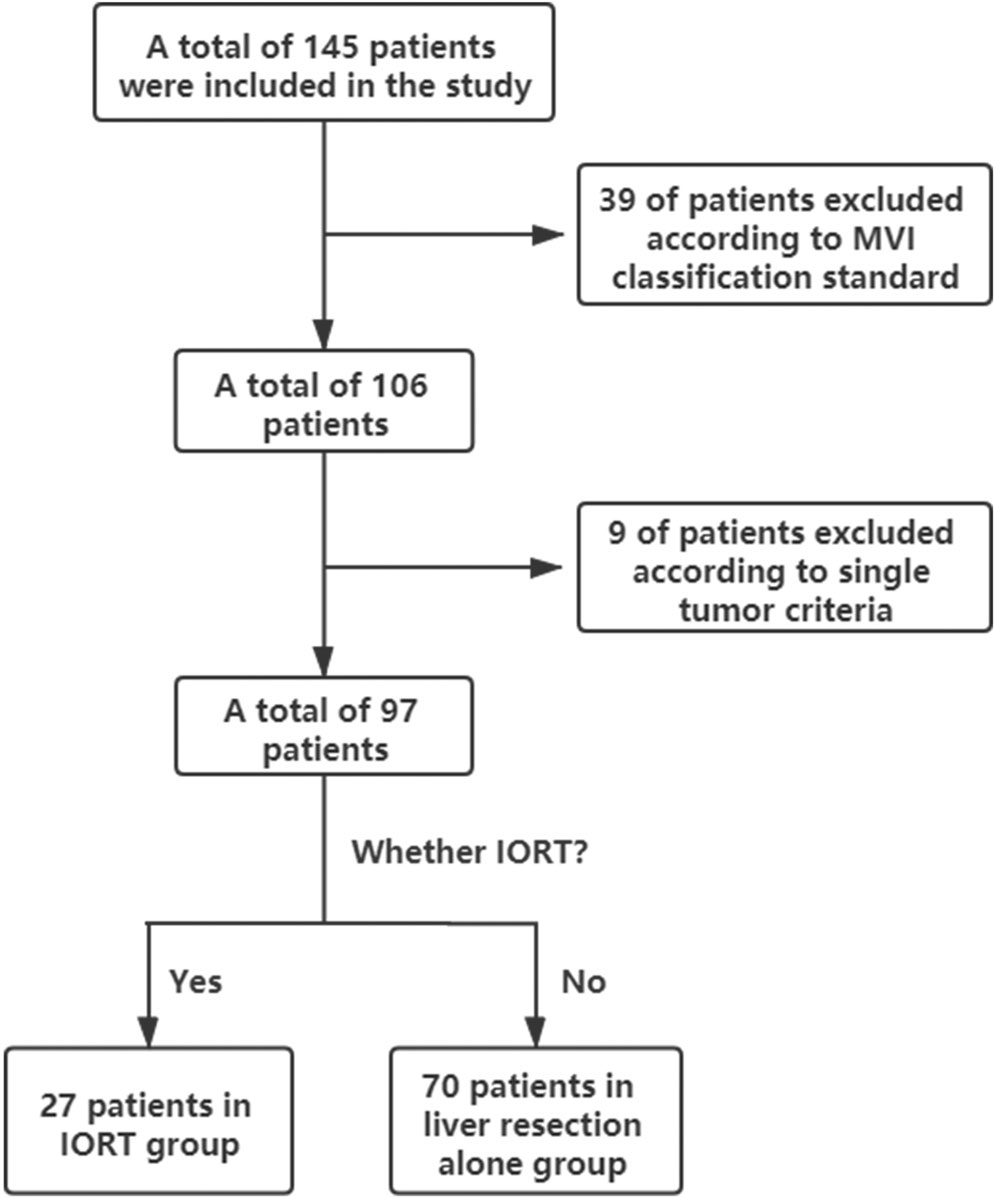
Flow chart for patient screening.

In total, 145 patients were initially enrolled based on predefined inclusion criteria. Subsequently, 48 patients were excluded from the study, including 39 with an indeterminate MVI grade and 9 with multiple tumours, in accordance with the predetermined exclusion criteria. A total of 97 patients were ultimately included and divided into two groups based on the application of IORT: IORT+LR (*n* = 27); and LR (*n* = 70). The patients in the IORT+LR group exhibited 1‐, 3‐, and 5‐year RFS rates of 66%, 50%, and 32%, respectively, whereas those in the LR group demonstrated rates of 54%, 37%, and 26%, respectively. The patient screening process in illustrated in Figure 1. The baseline characteristics of both groups before matching analysis are summarised in Table 1. A significant difference was observed between the two groups before matching based on standardised mean difference.

**TABLE 1:** Comparisons of baseline demographics and clinicopathological characteristics in patients undergoing LR + IORT or LR alone before and after propensity score matching analysis.

Characteristic	Before matching				After matching			
	LR + IORT (*n* = 27)	LR (*n* = 70)	Standardised mean difference^a^	*p*‐value	LR + IORT (*n* = 23)	LR (*n* = 23)	Standardised mean difference^a^	*p*‐value
Age (years)			0.36	0.109			0.09	0.753
≤60	16 (59.3%)	53 (75.7%)			15 (65.2%)	16 (69.6%)		
>60	11 (40.7%)	17 (24.3%)			8 (34.8%)	7 (30.4%)		
Sex			0.21	0.375			0.12	0.681
Male	24 (88.9%)	57 (81.4%)			20 (87.0%)	19 (82.6%)		
Female	3 (11.1%)	13 (18.6%)			3 (13.0%)	4 (17.4%)		
HBV			0.21	0.380			0.14	0.636
Positive	23 (85.2%)	54 (77.1%)			20 (73.0%)	21 (73.0%)		
Negative	4 (14.8%)	16 (22.9%)			3 (27.0%)	2 (27.0%)		
Liver function								
AST level	31.1 ± 10.1	34.5 ± 15.7	0.26	0.297	31.5 ± 10.6	33.2 ± 9.9	0.17	0.578
ALT level	32.8 ± 16.2	32.1 ± 15.0	0.04	0.844	34.0 ± 17.0	36.3 ± 15.4	0.14	0.632
TBIL level	13.3 ± 4.2	13.5 ± 5.9	0.04	0.855	13.1 ± 3.9	12.9 ± 4.1	0.05	0.864
Tumour								
AFP level^b^	1.80 ± 1.13	2.09 ± 1.33	0.23	0.322	1.72 ± 1.16	1.74 ± 0.85	0.01	0.961
MVI			0.09	0.690			0.09	0.760
M1	15 (55.6%)	42 (60.0%)			14 (60.9%)	15 (65.2%)		
M2	12 (44.4%)	28 (40.0%)			9 (39.1%)	8 (34.8%)		
Tumour size (cm)	5.33 ± 2.49	6.48 ± 3.15	0.41	0.091	5.40 ± 2.58	5.18 ± 3.27	0.07	0.807

*Note*: Variables are expressed as the mean ± SD(median with range) or *N* (%) (number with percentages), unless otherwise indicated.

^a^ Standardised differences of ≥0.1 represent meaningful differences in covariates between groups.

^b^ Variables are transformed as log10.

Abbreviations: AFP, a‐fetoprotein; HBV, Hepatitis B virus; IORT, intraoperative radiotherapy; LR, liver resection.

### 3.1 Propensity‐score matching analysis

**FIGURE 2:**
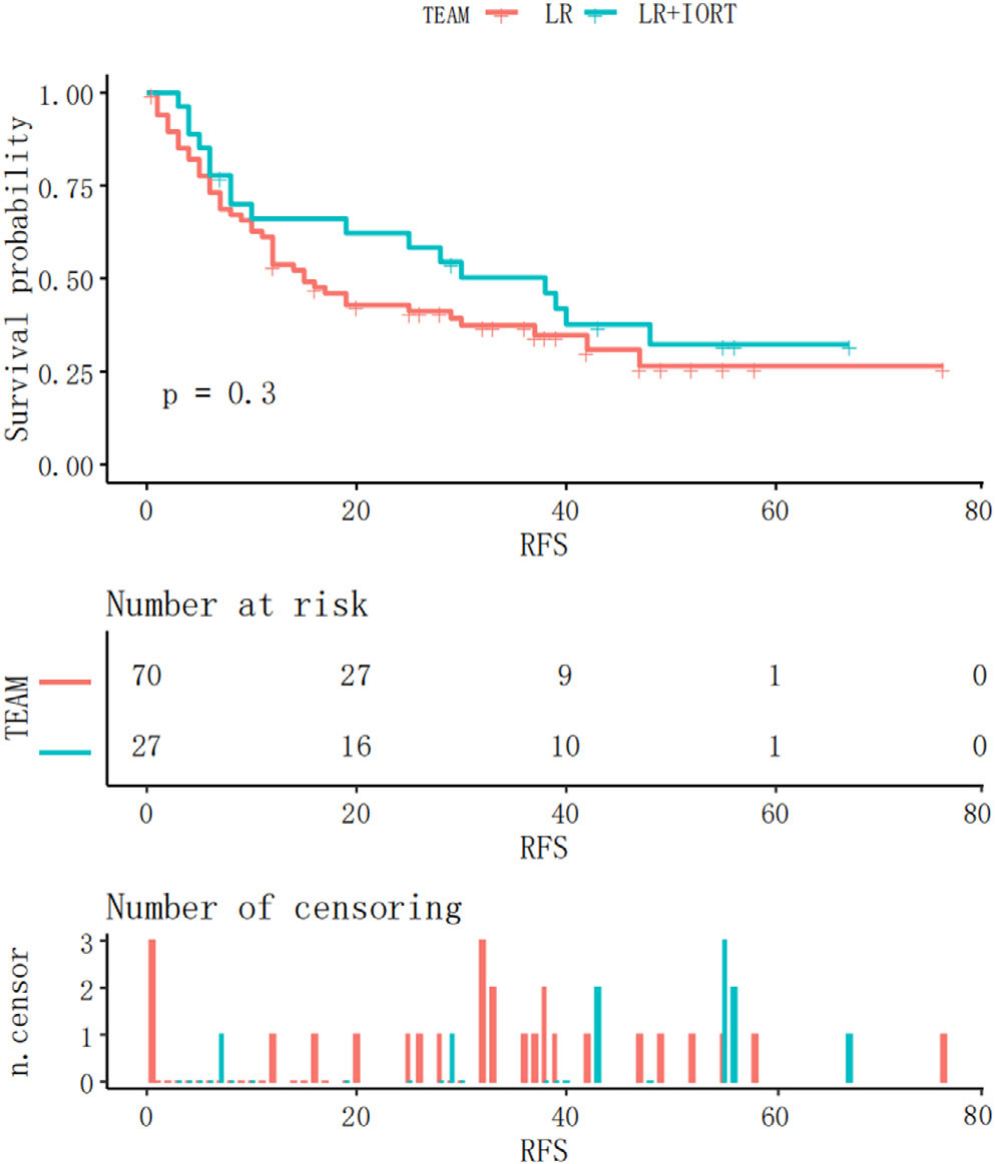
Kaplan–Meier RFS curves before matching in the IORT+LR and LR groups. IORT, intraoperative radiotherapy; LR, liver resection.

Kaplan–Meier analysis of RFS in the two groups (Figure 2) revealed no statistically significant difference (*p = *.3). To minimise selection bias, the 23 patients in the LR group were matched 1:1 with those in the IORT + LR group using matching analysis, which revealed no significant differences between the two groups (SMD <0.2, *p* > .05) (Table 1).

### 3.2 Survival benefits

**FIGURE 3:**
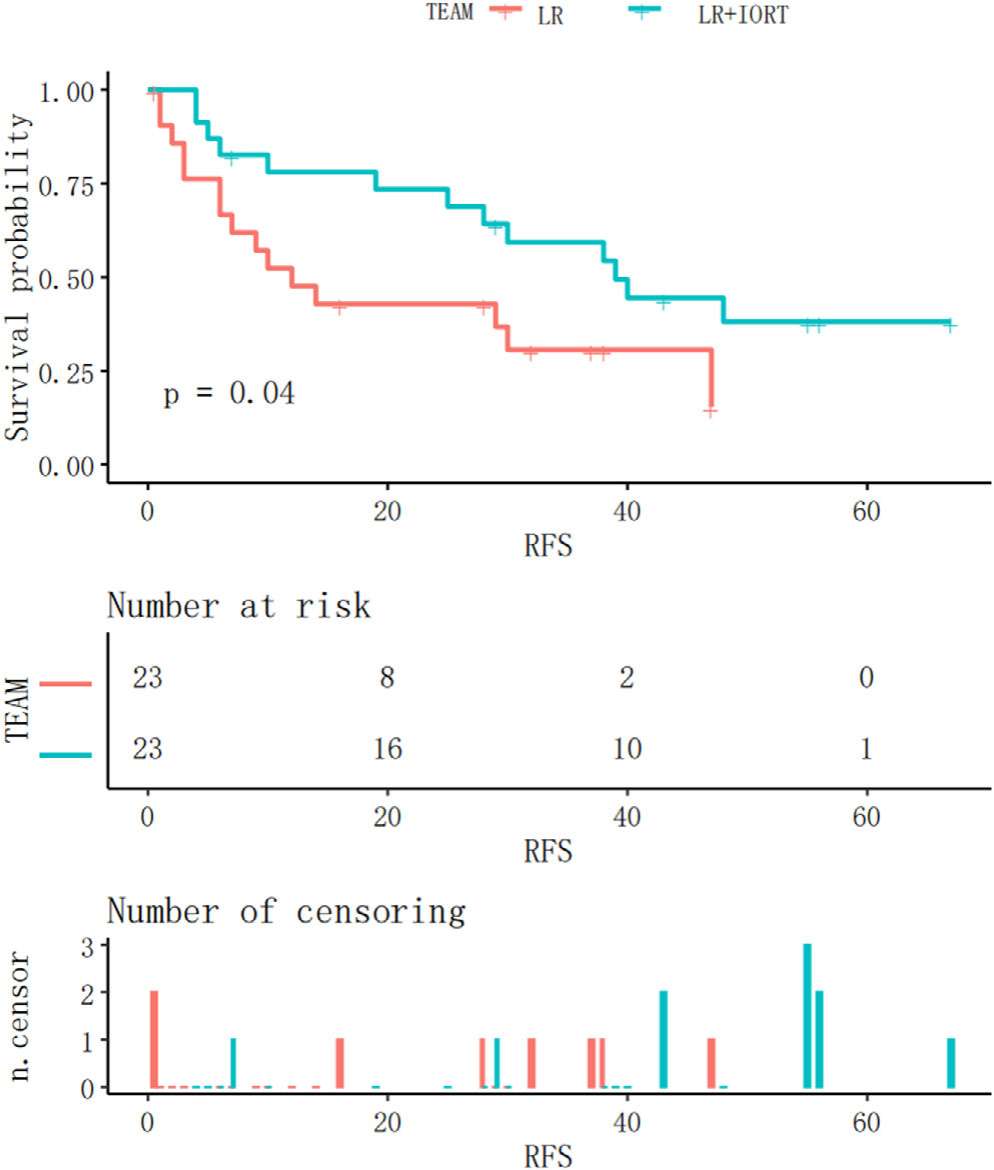
Kaplan–Meier RFS curves after matching in the IORT+LR and LR groups. IORT, intraoperative radiotherapy; LR, liver resection.

The Kaplan–Meier RFS curves after matching analysis for both groups are shown in Figure 3. The RFS of the IORT+LR group was significantly longer than that of the LR group after matching analysis (*p = *.04).

### 3.3 Cox regression

**FIGURE 4:**
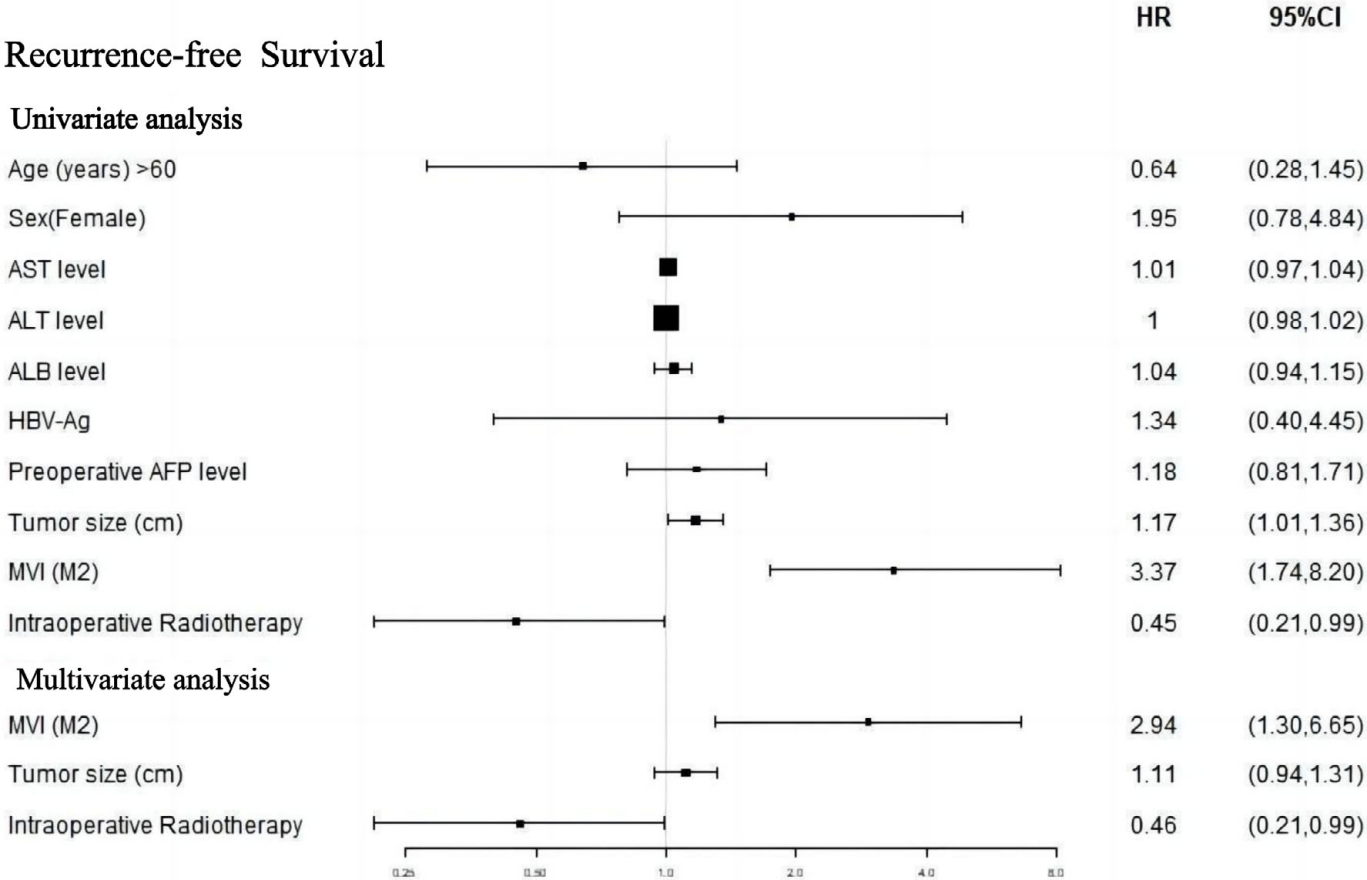
Cox proportional hazards regression for RFS.

The forest plot in Figure 4 demonstrates that in the univariate analysis, there was a notable correlation between IORT and improved RFS (HR 0.45 [95% CI 0.21–0.99]; *p = *.047). In the multivariate Cox analysis, three variables from the univariate analysis, namely IORT, MVI, and tumour size (*p* < .1), were incorporated. These findings indicate that both IORT and MVI (M2 grade) were independent prognostic factors for RFS.

### 3.4 Subgroup analysis in different MVI grades

**FIGURE 5:**
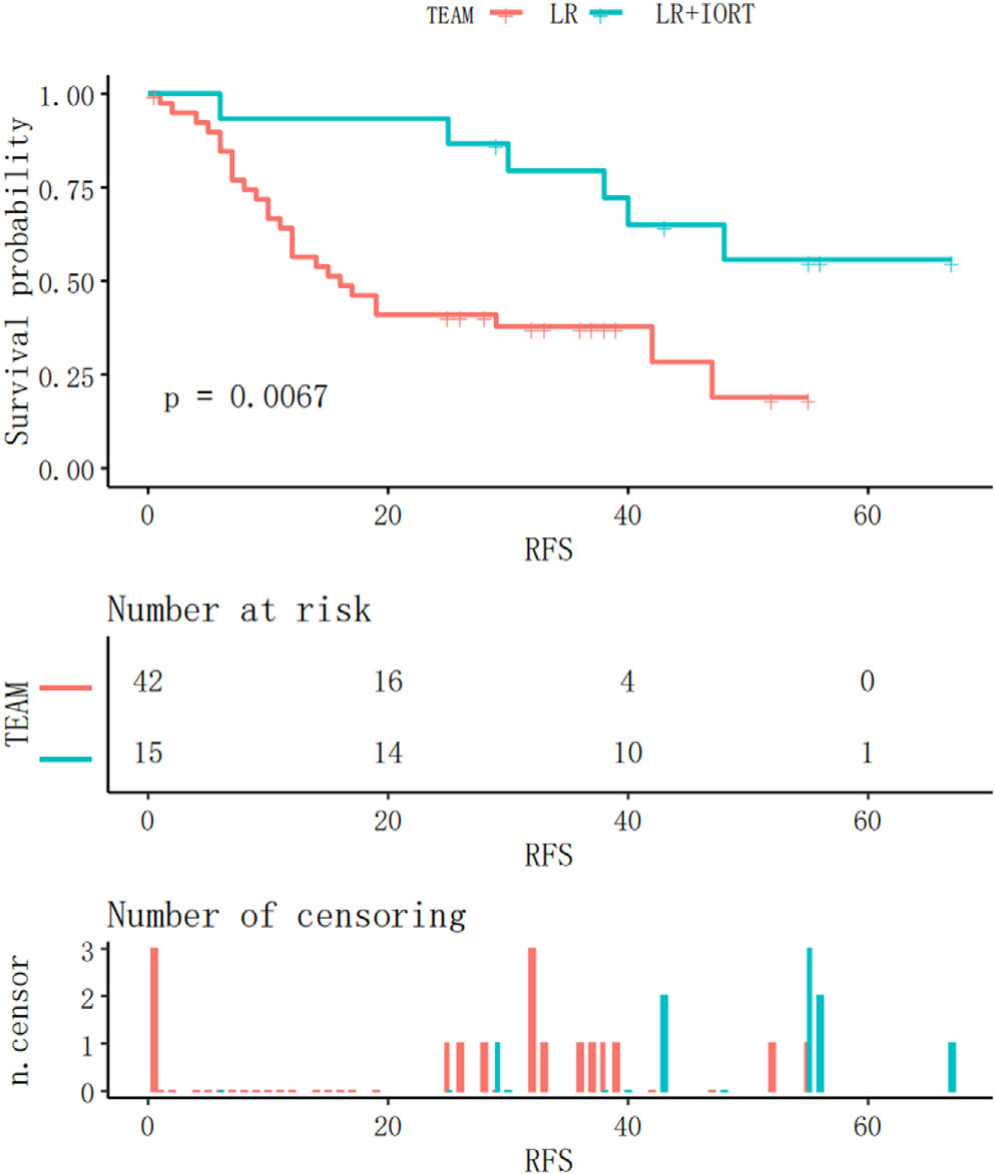
Kaplan–Meier RFS curves for M1 MVI patients in the IORT+LR and LR groups. IORT, intraoperative radiotherapy; LR, liver resection; M1, ≤5 microvascular invasion (MVI) areas, all located ≤1 cm away from the capsule of the tumour.

**FIGURE 6:**
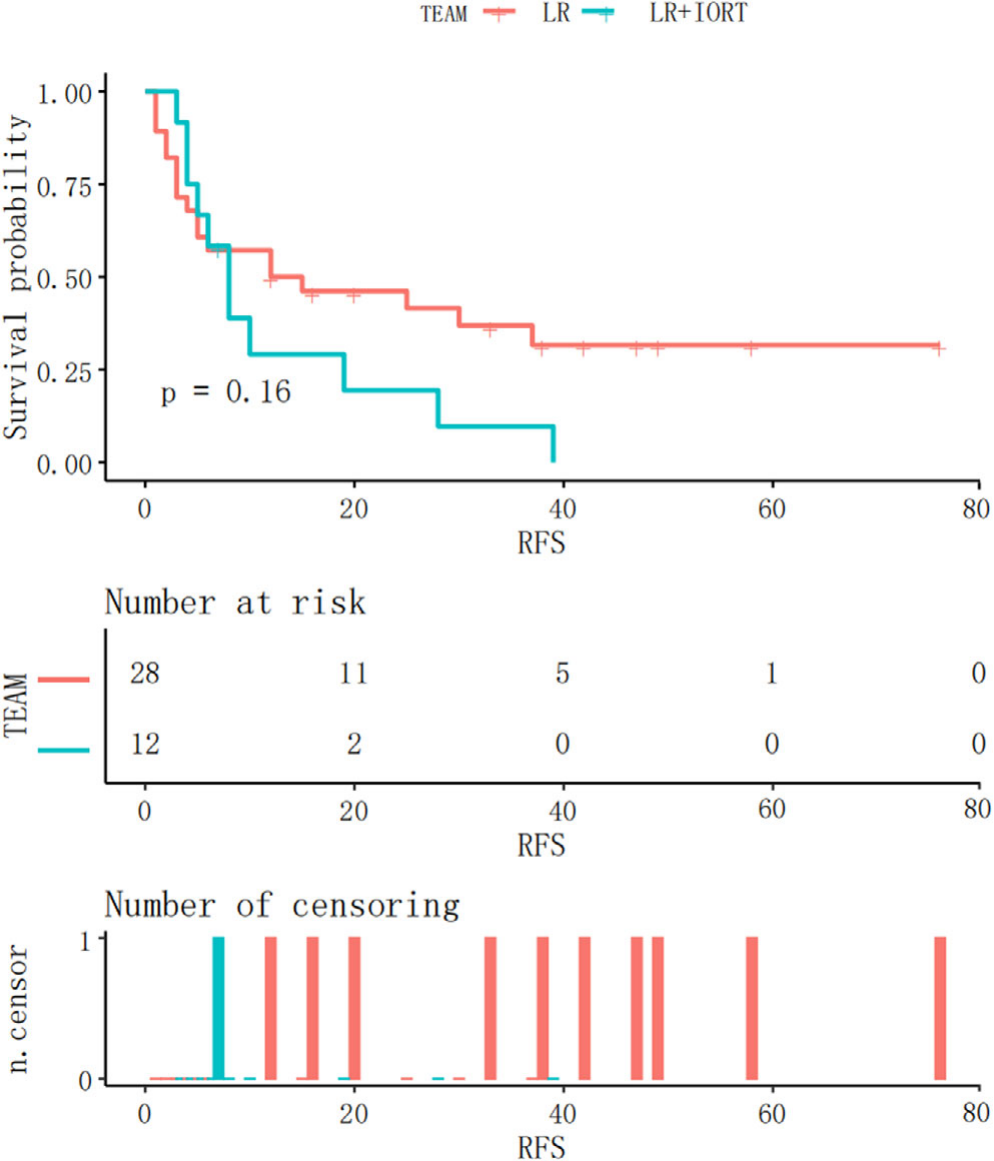
Kaplan–Meier RFS curves for M2 MVI patients in the IORT+LR and LR groups. IORT, intraoperative radiotherapy; LR, liver resection; M2, >5 areas of MVI or one of which were >1 cm away from the capsule of the tumour.

Subsequently, a subgroup analysis was performed to investigate whether RFS in patients with either M1 or M2 grade was significantly different between the two groups. In the IORT+LR and LR groups, MVI (M1 grade) was observed in 15 and 42 patients, respectively. The Kaplan–Meier RFS curves for patients with M1 grade are shown in Figure 5. RFS was significantly longer in the IORT+LR group than in the LR group (*p = *.0067) in patients with an M1 grade. MVI (M2 grade) was observed in 12 and 28 patients in the IROT+LR and LR groups, respectively. The Kaplan–Meier RFS curves before the matching analysis for patients with MVI (M2 grade) are shown in Figure 6. The findings indicated that there was no significant difference in RFS between the two groups of patients with M2 grade (*p = *.16).

### 3.5 Recurrence status and early recurrence analysis

This study included 97 patients, 61 of whom experienced recurrence. Among these, 17 were in the IORT+LR group and 44 in the LR group (Table 2). There were no significant differences in recurrence (*p = *.992). In the IORT group, 11 patients experienced intrahepatic metastases and six extrahepatic metastases. By contrast, the LR group included 34 patients with intrahepatic metastases and 10 with extrahepatic metastases. There was no significant difference in the number of patients with the two types of recurrences in either group (*p = *.344). A cut‐off point of 18 months^24^ was used to distinguish between early and late recurrences. In the M1 grade subgroup, the rate of early recurrence was significantly lower in the IORT+LR group than in the LR group (*p = *.003). In the M2 grade subgroup, there was no significant difference in the incidence of early recurrence (*p = *.505). Regarding recurrence location, the incidences of marginal and non‐marginal recurrence were 1 and 10 in the IORT group and 7 and 27 in the LR group, respectively. There was no significant difference between the two locations in either group (*p = *.657).

**TABLE 2:** Comparisons of recurrence in patients undergoing LR + IORT or LR.

	IORT+LR	LR	*p*‐value
Recurrence (Total)			0.992
Yes	17 /27	44/70	
No	10 /27	26/70	
Recurrence pattern (Total)			0.344
Intrahepatic recurrence	11/17	34/44	
Extrahepatic metastasis	6/17	10/44	
Early recurrence (M1 Group)			0.003
Yes	1/15	21/42	
No	14/15	21/42	
Early recurrence (M2 Group)			0.505
Yes	8/12	15/28	
No	4/12	13/28	0.657
Recurrence location			
Margin	1/11	7/34	
Non‐Margin	10/11	27/34	

*Note*: Variables are expressed as N/Total, unless otherwise indicated.

### 3.6 Sensitivity analysis suggests robust results

To assess the impact of unmeasured confounding factors on the primary findings, a sensitivity analysis was performed using an E‐value. The results were deemed robust, except in cases in which an unmeasured confounder had a relative risk of HR >2.80. The E‐value analysis indicated the robustness of the findings to unmeasured confounding factors.

### 3.7 Treatment process safety

All 46 patients underwent surgery without any safety concerns. No radiation‐related complications occurred in the IORT+LR group. There was no significant difference in mean (± SD) intraoperative bleeding and operative duration (>180 min) between the IORT+LR and LR groups (330.43 ± 216.24 vs. 580.4 ± 672.3 mL, *p = *.097; 95.7% vs. 87.0%, *p = *.295, respectively). Both groups experienced mild postoperative complications (91.3% vs. 82.6%; *p = *.381). Two patients developed postoperative biliary fistula and four developed postoperative pleural effusion, all of whom were treated with interventional therapy in both groups.

In summary, the IORT+LR group demonstrated significantly prolonged RFS compared with the LR group. IORT was identified as an independent prognostic factor of RFS. Furthermore, IORT is considered a secure and viable treatment alternative for patients with centrally located HCC.

## 4 DISCUSSION

The prognosis of patients with HCC after surgical resection is primarily influenced by postoperative tumour recurrence. However, it is commonly observed that a significant number of patients with HCC, particularly those located in the central regions, inevitably encounter tumour relapse after surgical resection, primarily attributed to the presence of a narrow incision margin measuring <1 cm or no margin at all. First, an insufficient margin leads to residual tumour cells, even if the postoperative pathology reports that the margin is negative (R0 resection). Second, early postoperative recurrence may have been caused by MVI around the tumour or the presence of satellite nodules. Hence, there is a need to develop a treatment strategy that is both efficacious and safe, with the aim of enhancing the prognosis of patients diagnosed with centrally located HCC and MVI. Our study is the first to provide retrospective, real‐world evidence that IORT is safe and effective for patients with HCC and MVI.

In this study, all patients had MVI. Some studies have suggested that MVI is the anatomical premise of HCC metastasis.^27^ MVI can be classified into M1 and M2 grades depending on the number of areas of MVI (< or >5)^28^ and the distance of these areas from the capsule of the tumour (< or >1 cm).^29^ Patients with a higher MVI grade have a poorer prognosis.^21^ Therefore, subgroup analysis was performed to investigate the efficacy of IORT for RFS in patients with M1 and M2 grades.

In M1 grade subgroup, IORT significantly reduced the incidence of early recurrence. Traditionally, the most intuitive advantage of IORT is that it reduces local recurrence caused by potential residual tumour tissue in the resection margin. It was also confirmed that IORT could reduce the incidence of margin recurrence, and only one patient in this group experienced this event.

IORT did not improve the RFS of patients with M2 grade, which may be due to several factors. First, the distance between the MVI and the capsule of the tumour was >1 cm, which was larger than the proposed intraoperative radiation exposure range, resulting in some tumour tissues avoiding radiation treatment, leading to an increased risk for postoperative tumour relapse, largely due to the emergence of new satellite nodules.^29^ Second, owing to a lack of references from other current studies, the radiotherapy regimen was established in a conservative manner to ensure patient safety, which may have resulted in an insufficient radiation dose, range, and time for IORT treatment in HCC patients with MVI.

Based on established consensus guidelines, the use of a conservative surrogate threshold effect of HR ≤0.6 for RFS serves as a reliable indicator of a significant improvement in OS.^30^ Our findings fulfil the aforementioned criteria and underscore the crucial role of IORT in improving the OS of patients with HCC following surgical intervention.

This study had some limitations, the first of which was its retrospective cohort design. As such, our findings require validation in a prospective study. Second, the sample size was limited, with only 97 patients included.

## 5 CONCLUSION

IORT+LR provided safe, feasible treatment for patients with centrally located HCC with MVI, along with an improvement in prognosis and lower early recurrence rates.

## AUTHOR CONTRIBUTIONS

**Zonggui Tao:** Investigation (equal); methodology (equal); writing – original draft (equal).

## FUNDING INFORMATION

This study was supported by Beijing Hope Run Special Fund of Cancer Foundation of China (LC2020L05).

## CONFLICT OF INTEREST STATEMENT

The authors have stated explicitly that there are no conflicts of interest in connection with this article.

## ETHICS STATEMENT

This retrospective non‐interventional study, which did not hinder diagnostic or therapeutic procedures, was approved by the Ethics Committee. The findings of this research will be disseminated in the form of statistically analysed data, ensuring the exclusion of any patient‐identifying information. All relevant patient data were kept confidential, in accordance with the Declaration of Helsinki. Consent was obtained from all participants.

## Data Availability

All data related to this study are included in this paper. Details are available from the corresponding author on reasonable request.
